# Adsorption of Polyphenols from Almond Blanching Water by Macroporous Resin

**DOI:** 10.1155/2022/7847276

**Published:** 2022-06-03

**Authors:** Malak Tabib, Christian Ginies, Njara Rakotomanomana, Adnane Remmal

**Affiliations:** ^1^Department of Biology, Faculty of Science Dhar El-Mahraz, University Sidi Mohammed Ben Abdellah, P.O. Box 1796, Fez 30050, Morocco; ^2^INRAE, Avignon University, UMR A 408, Green Extraction Team, F-84000 Avignon, France; ^3^INRAE, Avignon University, UMR408, Sécurité et Qualité des Produits d'Origine Végétale, Micronut Team, F-84914 Avignon, France

## Abstract

The almond processing industry generates large volumes of effluent after the blanching process. Blanching water is one of the main by-products with a potential source of polyphenols. However, before being used or discharged, this by-product requires pretreatment. This work was aimed at paving the way toward using adsorption on XAD-7 HP macroporous resin for wastewater treatment. This promising technique could be easily scaled up and integrated into existing production lines. Adsorption was carried out with a fixed bed in counterflow, while desorption was performed by acetone in downflow. With this approach, it was possible to concentrate up to five times the phenolic content of the initial blanching water. The resulting extract was analyzed by ultraperformance liquid chromatography-mass spectrometry (UPLC-MS), identifying more than 89% procyanidins, in addition to catechin, epicatechin, and isorhamnetin-3-O-rutinoside. Applications such as spray-drying and prilling techniques were suggested to improve the efficiency of polyphenols by preserving their stability, bioactivity, and bioavailability.

## 1. Introduction

The great production and transformation of almonds generate tons of waste yearly. Almond kernels are industrially separated from the skin by the blanching process performed by immersion in boiling water [1]. Subsequently, two by-products have received much attention: almond skins and blanching water, the latter being considered a great candidate to be a sustainable source of phytochemicals, such as flavonoids, tannins, and phenolic acids [2–4] Given the current legislation, blanching water must be disposed of as special waste, while the integuments are occasionally fed to animals as fodder [5]. Hence, the exploitation of almond by-products must be integrated into a sustainability context, outlining strategies to establish a circular economy around the almond industry [6].

Almond polyphenols can be recovered from blanching water [7] which is readily available and inexpensive. Resin adsorption has proven to be one of the most efficient techniques for the enrichment and recovery of polyphenolic plant secondary metabolites [8]. Resins are relatively simple in design and easy to regenerate, with a high capacity and favorable rate [9–10]. This technique is used for the treatment of organic substances and the concentration of polyphenols from wastewater (e.g., olive mill wastewater) and industrial streams [11]. Compared to conventional adsorbents such as silica gels, alumina, and activated carbons, macroporous polymer resins are considered more attractive alternatives due to their wide range of pore structures and physicochemical characteristics [12]. Furthermore, the adsorption processes can be easily scaled up and some resins are food-grade [13]. This technique provides many advantages, including the possibility of recycling and reuse, the absence of molecular cut-off, and applicability on an industrial scale [8]. The adsorption of almond polyphenols on resin has rarely been discussed in previous works. To our knowledge, the only published work that deals with the adsorption of blanching water polyphenols on the resin is the publication by Hellwig and Gasser [14], where they suggest the valorization of blanching water from marzipan production as a waste stream food industry. However, this review article does not describe all the materials and methods and does not give results of the yields obtained. It is therefore the first time that the methods, as well as all the characteristics of the extract obtained, have been described in their entirety.

As part of environmental awareness, a growing interest in waste stream valorization intended to fulfill the current global food demands was noticed. The present study introduces XAD-7 HP macroporous resin as an approach to recovering polyphenols from almond blanching water. After the determination of adsorption parameters, the dry extract obtained after acetone desorption and evaporation was analyzed by UPLC-MS to quantify the phenols retained during the adsorption process. Additionally, its antioxidant and antimicrobial activities were investigated. Finally, examples of applications such as atomization and prilling were suggested to ensure their immobilization, protection, and stabilization.

## 2. Material and Methods

### 2.1. Almond Blanching

Organic almonds (*Prunus dulcis*) were procured by Auchan (batch no. 2002M19, Avignon, France).

Almond blanching was performed by soaking 20 grams of almonds in 1 L of boiling distilled water (1 : 5 (*w*/*v*) ratio) for five minutes [7]. After draining the almonds, the blanching water (BW) was cooled instantly in an ice bath to room temperature, centrifuged twice at 9,000 rpm for 10 min (20°C) (SIGMA 4-16KS, Germany), and filtered in a Pyrex® filter funnel with sintered glass disc (porosity 4). The resulting BW was used successively without storing to avoid the risk of oxidation.

### 2.2. Resin Preparation

Amberlite® XAD-7 HP resin was purchased from Thermo Fisher Scientific (USA) [14]. According to the manufacturer, these acrylic beads have a surface area of 380 m^2^/g, a mean pore size of 300–400 Å, and a pore volume of 0.5 mL/g. Before adsorption, the resin was pretreated before use to remove impurities and pore blockage according to Sun et al. [15] method with slight modifications. Briefly, 300 grams of crude resin was rinsed six times with 100 mL of 95% ethanol and then with distilled water until the eluent was clear. Next, it was washed with 600 mL of 5% HCl and rinsed with distilled water until the pH of the eluent was neutral. Following this step, it was washed with 600 mL of 3% NaOH and rinsed with distilled water until the eluent was neutral. Finally, the resin with diethyl ether was dried using a rotavapor (Büchi Rotavapor R II, Switzerland) operated at 40°C.

### 2.3. Adsorption/Desorption Steps

The fixed-bed adsorption experiments were performed in XK 50 double-jacket glass column (England) with a length of 30 cm and an internal diameter of 5 cm. The column was filled with 150 g of preprepared resin to a height of 16 cm and then rinsed with distilled water to expand the resin. The polyphenols in the BW were adsorbed by upward flow using a peristaltic pump. The adsorption time lasted for 2 hours (until the concentration after adsorption is stable). The system has been connected with a nitrogen inlet to limit the oxidation of polyphenols.

For the desorption step, the resin was rinsed with acetone in a downward flow until clear fractions were obtained. Hence, the resin returns to its initial color. The volumes of acetone used were as follows: 250 mL for 1 L, 350 mL for 2 L, and 500 mL for 3 L and 4 L of adsorbed BW (HPLC grade, VWR Chemicals). Fractions were then collected in a round bottom boiling glass flask. Acetone was evaporated using a rotavapor operated at 40°C. The resulting dried extract was recovered and stored in hemolysis tubes at -20°C. The experiments ceased at the 4 L adsorption since equilibrium was reached as the mass extracted reached equilibrium.

The adsorption process is usually expressed by the adsorption capacity which represents the amount of solute retained per unit mass of the solid adsorbent as a function of the equilibrium concentration of the solute in the liquid phase at a constant temperature. Adsorption capacity *Q* (mg/g dry resin) at equilibrium was determined as given in the following equation [16]:

(1) $$\begin{array}{c} Q=\frac{\left(C0-C\text{e}\right)\;\times \;V\text{a}}{M}, \end{array}$$

where *C*_0_ and *C*_*e*_ are the initial and equilibrium concentrations of polyphenols in BW, expressed in mg/L, *V*_*a*_ represents the volume of BW used for adsorption in mL, and *M* is the dry weight of the resin used in g.

The specific surface adsorption capacity (SA, amount of bioactive compounds adsorbed per m^2^ of resin, equation (2)) was calculated as

(2) $$\begin{array}{c} \text{SA}=\frac{\left(C0-C\text{e}\right)\;\times \;V\text{a}}{M\times \text{SS}}, \end{array}$$

where SA is the surface adsorption capacity (mg/m^2^) and SS is the resin specific surface (m^2^/g) [13]. The same resin was recycled as described in Section 2.2 and reused for all experiments.

### 2.4. Free Radical Scavenging Activity (FRSA)

DPPH (2,2-diphenyl-1-picrylhydrazyl) is a highly colored reagent that allows fixing an H removed from the antioxidant contained in the extract with the concomitant formation of colorless hydrazine (DPPH-H) [17]. The FRSA assay was performed as described by Mimica-Dukic et al. [18] with some modifications. The obtained dry extract was dissolved in 80% (*v*/*v*) aqueous methanol to a final concentration of 10 g/L. In a microplate, 50 *μ*L of the extract solution was introduced into the microplate wells, serially diluted by half (from 10 g/L to 1.25 g/L), and then mixed with the same volume of a 0.5 mM DPPH solution in methanol. The absorbance was read at 520 nm using a FLUOstar Omega microplate reader (BMG LABTECH, Germany).

The percentage of inhibition was calculated from the following equation:

(3) $$\begin{array}{c} I\%=\frac{A0-\text{AE}}{A0}\times 100, \end{array}$$

where *A0* was the blank absorbance (without the extract) and AE was the absorbance of the extract at different concentrations [19]. The IC50 value (concentration giving *I*% = 50) was calculated by plotting *I*% as a function of the extract concentrations.

### 2.5. Ultrahigh Performance Liquid Chromatography

Polyphenol extracts were analyzed by ultraperformance liquid chromatography (UPLC) using an ACQUITY UPLC® system (Waters Corp., Milford, MA, USA) linked simultaneously to both a diode array detector 190–800 nm (UPLC DAD, Waters, Milford, MA, USA) and a mass spectrometer Bruker Daltonics HCT (High-Capacity Ion Traps equipped with an electrospray ion source (UPLC DAD/ESI-MSn)). Analysis was carried out using an ACQUITY UPLC® BEH C18 column (100 mm × 2.1 mm, i.d, 1.7 *μ*m; Waters Corp., Milford, MA, USA). The column temperature was set at 40°C. The injection volume was fixed at 2 *μ*L. The mobile phase was water containing 0.1% FA (formic acid) (solvent A) and 0.1% FA acetonitrile (solvent B) at the flow rate 0,4 mL/min. Linear gradient conditions were as follows: 0–6 min, linear gradient 97–90% A; 6–13 min, linear 90–70% A; 13–14 min linear 70–35% A; 14–15 min linear 30–0% A; 15–15.5 min linear 0-97%.

The ion trap was operated in the Ultra Scan mode from m/z 100 to 1500. The ICC target was set to 100,000 with a maximum accumulation time of 50 ms. Nitrogen (99.99% purity) was used as the desolvation gas. The ionization was achieved using an ESI source in negative mode. The ionization source parameters were set as follows: dry temperature 365°C, nebulizer pressure 50 psi, dry gas flow 9 L/min, and capillary voltage −2 kV.

The chromatographic peaks were tentatively identified by their MS and MS mass spectra and when this was possible, by injection of standards (catechin, epicatechin, vanillic acid, chlorogenic acid, isorhamnetin, and kaempferol). Quantification was performed with UV signal on the basis of the calibration curve of catechin, epicatechin, and vanillic acid at 280 nm, chlorogenic acid at 330 nm, and isorhamnetin and kaempferol at 360 nm, using Bruker Daltonics software (Massachusetts, United States).

Other compounds for which standards were not available (procyanidins and heterosides) are expressed as equivalent to the injected standards: (epi)catechin oligomers with catechin,and 4-p-coumaroylquinic acid with coumaric acid, vanillic acid oligomers with vanillic acid, and kaempferol and isorhamnetin oligomers with kaempferol and isorhamnetin.

#### 2.5.1. Polyphenol Content

A known mass of resin-obtained extracts (ROE) and freeze-dried extracts (FDE) from BW was dissolved in 500 *μ*L of an 80% methanol solution. The mixtures were vortexed and sonicated for 5 minutes. After centrifugation, supernatants were collected and then diluted twice with distilled water before injection.

#### 2.5.2. Procyanidin Content

Procyanidins in the two extracts were evaluated as follows: briefly, known masses of the ROE and FDE were added to 600 *μ*L of a menthofuran solution (6 g/L in methanol) and 200 *μ*L of an anhydrous HCl solution (0.4 M in methanol). The solutions were incubated for 1 hour at 40°C under continuous stirring and then injected for analysis.

Procyanidin concentration (PC) in almond skin was calculated using the following equations [20]:

(4) $$\begin{array}{c} \text{PC}=\left({\left[\text{upper and extension units}\right]}_{M}\;+{\left[\text{end units}\right]}_{M}\right), \end{array}$$

(5) $$\begin{array}{c} \text{PC}=\left(\left[{\text{cat}}_{\text{MF}}\;\right]-\left[{\text{cat}}_{C}\right]\right)+\left(\left[{\text{epicat}}_{\text{MF}}\right]-\left[{\text{epicat}}_{C}\right]\right)+\left[\text{cat}-\text{MF}\right]+\left[\text{epicat}-\text{MF}\right], \end{array}$$

where [cat_MF_ ] and [epicat_MF_] are the concentrations of catechin and epicatechin after menthofuranolysis, [cat_*C*_] and [epicat_*C*_] are the concentrations of crude catechin and epicatechin before treatment, and [cat − MF] and [epicat − MF] are the concentrations of catechin-menthofuran and epicatechin-menthofuran, respectively. The mean degree of polymerization (mDP), i.e., the average number of units in the polymer, can also be determined in addition to the concentration of procyanidins. mDP of the extract was calculated according to equation (6):

(6) $$\begin{array}{c} mDP=\frac{{\left[\text{upper and extension units}\right]}_{M}+{\left[\text{end units}\right]}_{M}}{{\left[\text{end units}\right]}_{M}\;}. \end{array}$$

### 2.6. Antimicrobial Activity

The antimicrobial activity of the extract was evaluated against two bacterial strains: *Escherichia coli* ATCC 25922 (Gram -) and *Staphylococcus aureus* ATCC 29213 (Gram +). The minimal inhibitory concentration (MIC) was determined using a 96-well plate according to the standards of the CLSI [21]. Ten concentrations of the extract were prepared using sterile distilled water and were between 30 mg/mL and 0.25 mg/mL.

Bacterial suspensions were prepared as follows: briefly, stock cultures that were kept in a Mueller–Hinton agar (MHA, Biokar®) at 4°C were suspended in sterile saline solution (9 g/L NaCl). After shacking, their density was adjusted to the turbidity of a McFarland standard of 0.5 (equivalent to 1.5 × 10^8^ CFU/mL) [22]. The concentrations of the extract previously prepared were diluted with appropriate volumes of Mueller–Hinton broth (MHB, Biokar®) and introduced into the 96-well plate with the bacterial cultures. The plates were afterward shaken for 10 minutes in an IKA® MS 3 digital shaker (China) and then incubated at 37°C for 24 hours. The two experiments, in addition to a blank, were performed in duplicate. The MIC corresponds to the lowest concentration that does not show bacterial growth.

### 2.7. Applications

#### 2.7.1. Prilling/Encapsulation

Alginate capsules and beads were produced with high-frequency vibration apparatus. A Büchi B-395 Pro (Switzerland) encapsulator was used to set up process parameters to obtain uniform size beads/capsules to encapsulate almond extract solutions. Two types of nozzles were used, including a single nozzle for beads and a concentric nozzle for aqueous-core and oil-core capsules. The operating settings of the Büchi B-395 Pro have set values for voltage (250-2500 V), vibration frequency (40-6000 Hz), air pump system (0.5-200 mL/min), and syringe pump system (0.01-50 mL/min).

Three different products were obtained:

- Beads: a solution of 1 g/L of REO was prepared in a 1.5% (*w*/*w*) low viscosity grade sodium alginate solution in osmosed water (Büchi, Switzerland). A single nozzle with a size of 1000 *μ*m was used. After degassing the setting and adjusting the frequency, pressure, and flow parameters, the solution was sprayed in a 150 mL beaker containing 75 mL of a 5% calcium chloride solution (Merck) under continuous stirring
- Oil-core capsules: a solution of 0.2 g/L was prepared after dissolving a mass of the extract in sunflower oil. After sonication, the core solution was filled into a syringe and connected to the pulsation chamber. This same chamber was linked to a pressure bottle containing the shell, a 2% (*w*/*w*) sodium alginate solution previously degassed. A 150 mL beaker was filled with 5% (*w*/*w*) calcium chloride and placed under the nozzle on the magnetic stirrer. Depending on the diameters of the internal and external diameters of the nozzles, the parameters of the encapsulator were set to obtain an accurate microparticle chain in the light of a stroboscope lamp
- Aqueous-core capsules: a solution consisting of the extract at 1 g/L was used as the core solution. The pressure bottle was filled with a degassed solution of 2% sodium alginate with SDS at 10 mM. The beads were washed with 5% CaCl_2_ and 0.1% Tween 20. The same parameters previously mentioned were modified to have uniform size capsules according to the diameters of the internal and external nozzles

A release test of the capsule contents was performed on the beads obtained by the 1000 *μ*m nozzles. For this purpose, a solution of pH 5 was obtained by mixing 2.57 g of sodium citrate and 0.24 of citric acid in an aqueous solution (osmosed water). The pH variations (adjusted to 3, 5, and 12) were achieved by adding 0.1 M HCl and 0.1 M NaOH solutions. In addition, the destructive effect of a 1 M NaCl solution was tested on the beads.

#### 2.7.2. Spray-Drying–Atomization

The spray-drying equipment consisted of a Büchi Mini Spray-Dryer B-290. A 200 mL aqueous feed solution composed of 16% maltodextrin, 20% acacia gum, 0.1% ROE, and 1% of essential oil (e.g., *Citrus limonum* essential oil) was prepared using a magnetic stirrer. The operating variables of the process were selected based on the results of several runs carried out with similar samples [23].

The feed solution is transferred by a peristaltic pump through a nozzle to the drying chamber previously heated at an inlet temperature of 120°C. The dried particles are then led to the collection vial by cyclone technology. Compressed nitrogen was used as the atomizing gas while conditioned room air served as the inlet [24].

## 3. Results and Discussion

### 3.1. Adsorption/Desorption on Resin

Table 1 illustrates the average concentrations of the extracts in the three volumes of blanching water before and after adsorption as well as the masses of the extract obtained. It can be observed that the adsorption of almond blanching water enables its concentration up to five times, where the concentration noticeably increases from an average of 1.1 g/L of BW to about 5.9 g/L of acetone. The XAD-7 HP resin is therefore an effective technique for the concentration of almond blanching water, considered a diluted system.

The equilibrium was reached after the adsorption of 3 L of BW. The difference between *C*_*f*_ corresponding to 3 L and 4 L is not significant.

**Figure 1:**
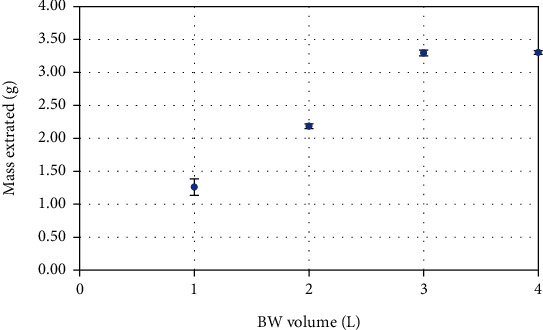
Masses extracted after adsorbing 1 L, 2 L, 3 L, and 4 L of blanching water, expressed in g.

Figure 1 confirms the results presented in Table 1. It shows a steady increase in extract mass after adsorption of 1 L to 3 L of BW volumes on the resin and stabilizes after adsorption of 4 L of BW. Complete evaporation of acetone resulted in a dark brown powder that was collected and then stored in a glass container at −20°C for further analysis.

According to equations (1) and (2), the adsorption capacity (*Q*) of the resin is 21 ± 0.29 mg/g resin, and the surface adsorption capacity (SA) is around 0.06 mg/m^2^.

Alongside the results described above, the effectiveness of macroporous resin as an adsorbent of polyphenols in effluents was described in many recent studies. An example of olive mill wastewater (OMW) adsorption on resins has been widely studied. The results in Vavouraki et al. paper [11] showed that resins represent an effective method for polyphenol recovery from OMW. A reduction of 75% of the soluble phenolic content was observed when FPX 66 resin was used. Another statement in Thi Le et al. paper [13] showed that XAD7 had a better surface adsorption capacity (0.027 ± 0.00146 mg/m^2^) than XAD16 resin for the adsorption of phenolic compounds from an aqueous by-product of sunflower protein isolate production. This result is almost similar to the one obtained in this study, considering the specific surface (450 m^2^/g versus 380 m^2^/g) and the type of phenolic compounds to be adsorbed (phenolic acids compared to flavonoids). Another study suggested an alternative methodology to manufacture value-added products from green coconut fiber [25]. The findings of this paper showed that adsorption using XAD-7HP resin allowed the recovery of bioactive compounds, including catechin, epicatechin, and condensed tannins.

### 3.2. Identification of Phenolic Compounds in Almond Skin

#### 3.2.1. Qualification of Phenolic Compounds in Almond Skin Extracts

**Figure 2:**
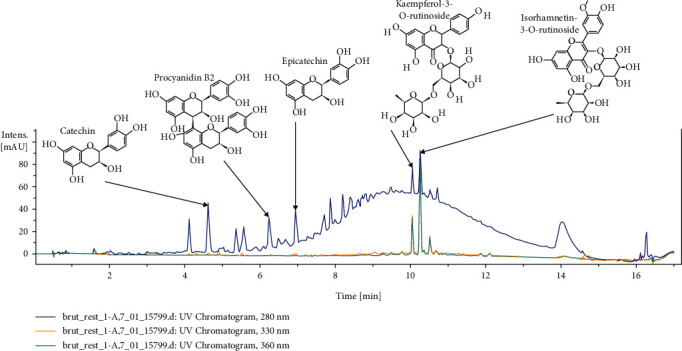
UPLC chromatogram of phenolic compounds in almond skins.

Figure 2 shows the UPLC chromatogram of the phenolic compounds from almond skins. A total of 20 components, including flavanols, flavonols, flavanones, and nonflavonoids were identified (Table 2), based on their retention time and their UV and mass spectra.

As reported by others [7, 14], the most abundant phenolic components were identified by matching the retention time and their average absorbance (280 nm for vanillic acid and flavanols, 330 nm for chlorogenic acid, and 360 nm for flavonols).

Flavanols were the major almond flavonoids identified, including catechin and epicatechin as monomers, procyanidins as polymers (B-type), and oligomers. Catechin and epicatechin were detected at 4.6 min and 7 min with a molecular ion ([M-H]^−^) at m/z 289 and correspond to peaks 11 and 16, respectively. As for catechin derivates, (epi)catechin digallate and (epi)catechin di-hexose corresponded to peaks 1 and 8 with a molecular weight of 593 and 613, respectively.

Vanillic acid was also identified linked to one or two hexoses (glucose and/or galactose; m/z 329 and 491). Flavonols were identified in peaks 17 to 20 and corresponded to kaempferol (as -3-O-rutinoside and -3-O-glucoside) and isorhamnetin (as -3-O-rutinoside). 3-O-caffeoylquinic was found at 2.9 min, while chlorogenic acid was detected at 4.75 min (m/z 353).

Peaks 2, 10, and 21 belong to protocatechuic dihexose, 4-p-coumaroylquinic acid, and rutin (m/z 477, 337, and 609). These results are in agreement with the findings of our previous study [7]. In another paper, Hughey et al. confirmed the presence of the compounds cited above, including catechin, epicatechin, isorhamnetin and its oligomers, kaempferol and its oligomers, and procyanidins [26].

#### 3.2.2. Quantification of Phenolic Compounds in Almond Skin

Table 3 sums up the concentration of the phenolic compounds identified in ROE and FDE, expressed in mg/kg of almonds.

The same compounds presented in Table 2 were added, including flavanol polymers (dimers, trimers, and tetramers) that were accounted for as procyanidins.

Procyanidins represent the majority of compounds of both extracts (more than 89%), followed by catechin, epicatechin, and isorhamnetin-3-O-rutinoside. Procyanidin quantification was carried out using menthofuranolysis. Menthofuran can depolymerize procyanidins, to allow their characterization (average degree of polymerization) and to quantify them as can be done by thiolysis without the inconvenience of a nauseabond odor hardly compatible in a laboratory [27]. Besides the concentration, menthofuranolysis enables the determination of the mean degree of polymerization (mDP) which was approximately 6 for both extracts. This result is perfectly consistent with the findings of a previous study [7], where procyanidins represent over 85% of the total extract. Product analysis after menthofuranolysis showed four major products, including catechin, epicatechin, catechin-menthofuran, and epicatechin-menthofuran.

Although similar qualitative profiles have been reported in previous studies [2, 28, 29], it is not possible to make meaningful comparisons due to differences in variety, year of production, and environmental conditions [30].

An increase in flavonoid concentrations was observed for ROE compared to FDE. We suggested that the chalcone nuclei of the flavonoids that occur naturally in almond skin equilibrate spontaneously. Therefore, it undergoes cyclization under neutral conditions as almond blanching water cycles for 2 hours in the adsorption column (Figure S3) [31]. This leads to an increase in the concentration of catechin, epicatechin, and their oligomers, as well as kaempferol and isorhamnetin. In their study, Aneklaphakij et al. [32] confirmed the presence of chalcones in almond kernel skins. In addition, Khan et al. [33] demonstrated that naringenin and hesperetin chalcones spontaneously undergo cyclization back to the parent flavanones under neutral conditions.

### 3.3. Antioxidant Activity

The antioxidant potential of the extract was evaluated. Phenolic antioxidants are typically able to quickly reduce reactive oxygen species (ROS), including free radicals, thereby protecting biomolecules (e.g., polyunsaturated fatty acids) against oxidation [34]. IC50 represents the concentration of Trolox® that allows 50% of the radicals present to be trapped [35].

In the present study, the four concentrations discolored the DPPH. The IC50 value of the extract was 2.905 mg/mL, equivalent to 7.384 g Trolox eq/g dry extract.

Wood et al. [36] stated in their study that procyanidins exhibit an interesting antioxidant effect under acidic and basic conditions, but a moderate effect in neutral solutions. This may explain the high value of IC50 for the extract since procyanidins represent almost 90% of the total extract. Moreover, the antioxidant power of procyanidins is not high according to the DPPH method. However, when ingested, the absorption of these products is facilitated at the level of the intestine. Therefore, their antioxidant power is much higher because, that over time, these condensed tannins are hydrolyzed into catechins and epicatechins, hence the added value of these extracts [37].

### 3.4. Antibacterial Activity

After 24 hours of incubation, it was noticed that the extract did not affect the bacterial growth of *E. coli*. On the other hand, an interesting effect was noticed for *S. aureus*, as the extract inhibited their bacterial growth with a MIC of 2 mg/mL. 50 *μ*L volumes were sampled from the wells containing MIC, MIC x2, and MIC x4 to determine the bactericidal/bacteriostatic effect of the extract. It was noticed that no growth occurred using the MIC alone, proving that this dose is bactericidal.

As reported by Mandalari et al. [1], blanched almond skin flavonoid-rich extract had a bacteriostatic inhibitory effect at 0.5 mg/mL for *Staphylococcus aureus* ATCC 6538P strains. These results are in agreement with the results obtained above, noting that this extract has an impact on the growth of Gram + bacteria, but no significant effect on Gram - bacteria.

### 3.5. Applications

Contemporary industrial food production involves the addition of functional ingredients to adjust flavor, color, texture, or preservative properties. The latest trend is the inclusion of bioactive compounds extracted from natural sources with potential health benefits, such as antioxidants and probiotics [38].

The main objectives of using polyphenol encapsulation techniques are to provide protection and stabilization of an active compound, as well as its controlled release using a natural polymer [39, 40]. Indeed, these are critical parameters for their successful incorporation into various food systems, in addition to their controlled release in the gastrointestinal tract, as their submission and rapid first-pass metabolism lead to chemical structure transformation and changes in their bioactivities [38].

#### 3.5.1. Prilling-Encapsulation

The main objectives of using polyphenol encapsulation techniques are to ensure the protection and stabilization of an active compound, as well as its controlled release using a natural polymer. Indeed, these are critical parameters for their successful incorporation into various food systems, in addition to their controlled release in the gastrointestinal tract as their submission and rapid first-pass metabolism results in the transformation of the chemical structure and changes in their bioactivities [40].

**Figure 3:**
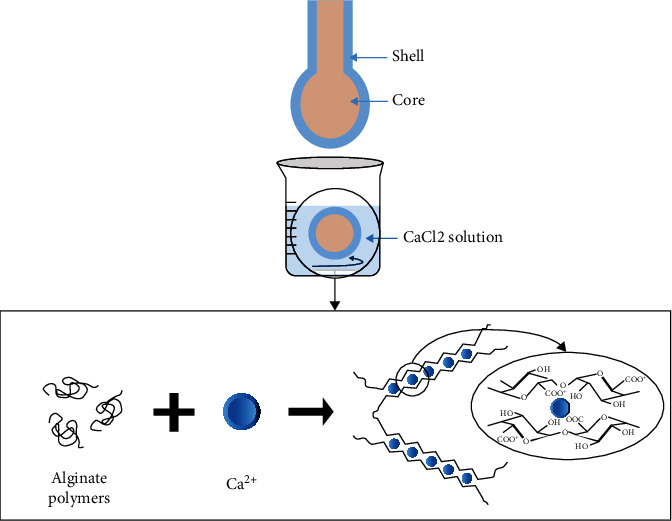
Capsule production process. A capsule is formed by coextrusion of the core solution (water or oil) and the alginate solution. When dropped into a calcium bath, the alginate instantly gels to form a capsule with a liquid core and a hydrogel membrane [39, 41, 42].

In this study, three types of capsules were produced using Encapsulator B-395 Pro. For the alginate-extract beads, various nozzles were used. It was noticed that only the beads obtained with a 1000 *μ*m nozzle were stable. An increase in CaCL_2_ bath concentration from 1.47% to 5% was also mandatory to slow down the extract release (Figure 3).

**Figure 4:**
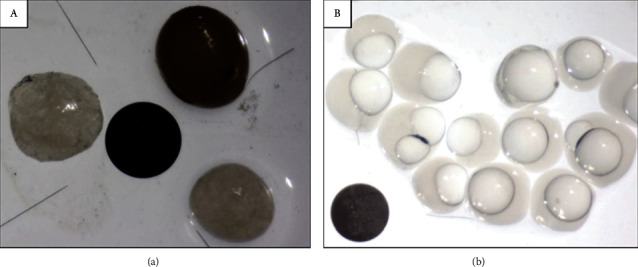
Observation under binocular magnifier. (a) Single alginate bead (dark) and two aqueous core capsules of different nozzle sizes (450-900 *μ*m and 300-500 *μ*m) compared to the center (black disk) of size 1.5 mm. The diameter of the capsules is around 2 mm, 1.84 mm, and 1.45 mm, respectively. (b) Oil-core capsules obtained with 450-600 *μ*m nozzle sizes. Sizes are around 1.45 ± 0.21 mm.

Oil-core capsules were successfully obtained with various nozzle sizes after setting the frequency and pressure parameters, including 120 *μ*m–300 *μ*m, 150 *μ*m–300 *μ*m, 200 *μ*m–300 *μ*m, 300 *μ*m–500 *μ*m, and 450 *μ*m–600 *μ*m (sizes of the core and shell nozzles, respectively) (Figure 4).

Aqueous-core capsules were obtained according to the method described by Doméjean [41] with the same nozzle sizes used for oil-core capsules. Since the core and the shell are aqueous solutions, the addition of SDS is important for the stability of the capsules. The formation of the precipitate with CaCl2 confers a certain rigidity to the capsule membrane which limits the deformation and mixing of the fluids at impact and thus facilitates the formation of the capsule. In addition, like Tween 20, SDS also promotes wetting of the capsule within the CaCl2 bath.

Further tests were conducted on the reversibility of the capsules and the release of their phenolic content. Indeed, a destructive effect was observed after 10 minutes and 24 minutes of pH adjustment to 5 and 12, respectively. On the other hand, the release time of the bead contents at pH 3 is considerably long (more than 45 minutes). An interesting but partial effect was noticed after the addition of NaCl solution only, leaving alginate particles in suspension.

Up to date, encapsulation of functional food ingredients/bioactive compounds is still performed at low levels (1-5%), as the maximum acceptable cost of an encapsulation process is rather low, 0.1 €/kg of new product according to Đorđević et al. report [38]. Hence, an increasing number of food manufacturers are proposing to improve the encapsulation process and study the feasibility of scaling up. The French company Capsulæ, for instance, offers the possibility of scaling up the microencapsulation process to an industrial scale. It provides design solutions and solves encapsulation issues to generate commercially viable techniques with a wide spectrum of product applications.

To conclude on the prilling technology, the capsule or “hydrogel” forms are reversible since the network is a polymer tangle held by secondary forces such as hydrogen, ionic, or hydrophobic bonds. Alginate remains an ideal candidate for biomedical and biotechnological applications [42]. Moreover, its gelation in the presence of calcium ions is simple to implement and instantaneous and allows to form a relatively rigid physical hydrogel. The drawbacks of this technique lie in the low solubilization of the extract in the oil and the limited stability of the bead with small diameters.

#### 3.5.2. Spray-Drying–Atomization

The spray dryer parameters that were applied in the study are as follows: the inlet and outlet temperatures were set at 120°C and 68°C, the aspirator and the sample pump were fixed at 100% and 30%, and the airflow rate was adjusted to 40 mm. The mass of the resulting product was 20.656 g.

Spray-drying technique is an efficient technique for the encapsulation of polyphenols with maltodextrin as a carrier [43], as it is flexible, operates continuously, and produces good quality particles. It is widely used in the food industry for food additives and flavors [40].

The spray-drying process is easily industrialized. It is based on a well-established technology of relatively low cost, which requires equipment that is already used in industrial sectors (food, pharmaceutical, and chemical production). Indeed, the scaling-up from the laboratory to production requires the control of many factors, including the formulation (concentration of active compounds and coating materials, viscosity, etc.), inlet temperature, and flow rate.

Spray-drying has proven to be a popular technique for the protection of phenolic compound activities in various wall materials [44, 45]. In a study, researchers have highlighted the importance of spray-drying in the preservation of antioxidant and antimicrobial activity of polyphenols of olive leaf extracts in processed food [46].

## 4. Conclusion

The adsorption of the bioactive compounds on the XAD-7 HP resin was found to be a very favorable process for the purification of almond blanching water. The methodology allowed the concentration of the phenolic content of the blanching water to increase more than 5 times. These results may lead to a potential scale-up for polyphenol recovery in the almond industry since it has been proven to be a cost-effective method [47]. The obtained extracts showed high concentrations of condensed tannins, catechin, epicatechin, and isorhamnetin-3-O-rutinoside. They have also demonstrated an interesting antibacterial effect on *Staphylococcus aureus* ATCC 29213. These recovered fractions can be used in the food industry, in cosmetics, or in pharmaceuticals in the forms presented previously, including capsules, beads, and atomized extracts that can be easily incorporated into preparations. As a matter of perspective, the adsorption process can be scaled up to recover phenolic compounds of high interest in the context of green chemistry heading towards renewable resources. The incidence of solvent consumption for elution of compounds retained by XAD-7 should be considered relevant when scaling up the process.

## Figures and Tables

**Table 1:** Concentration of the extracts in three volumes of blanching water (1-3 L) before adsorption expressed in g/L of BW and after adsorption expressed in g/L of acetone.

BW (L)	*C*_0_ (g/L of BW)	*C*_*f*_ (g/L of acetone)
1	1.260 ± 0.126	5.040 ± 0.306
2	1.092 ± 0.036	6.240 ± 0.103
3	1.098 ± 0.044	6.588 ± 0.088
4	1.160 ± 0.113	6.605 ± 0.056

**Table 2:** Phenolic compounds identified by UPLC-DAD/ESI-MSn in almond skin extracts.

Peak	Suggested structure	*t*_*R*_ (min)	[M-H]^−^ (m/z)	MS2 (m/z)	UV_max_ (nm)
1	(Epi)catechin digallate	1.5	593	441; 315; 289	277
2	Protocatechuic dihexose	1.9	477	323; 225; 153	277
3	Vanillic acid dihexose	2	491	323; 167	256; 288
4	Vanillic acid hexose	2.2	329	167	254; 291
5	Vanillic acid dihexose	3	491	323; 167	279
6	3-O-caffeoylquinic	2.9	353	191	211; 344
7	(Epi)catechin dihexose	3.2	613	289	280
8	B-type dimer	4	577	289	280
9	4-p-Coumaroylquinic acid	4.2	337	163; 191	308
10	Catechin	4.6	289	245	280
11	Chlorogenic acid	4.75	353	191; 179	325
12	B-type trimer	5.3	865	577	280
13	B-type tetramer	5.6	1153	289; 865	280
14	B-type dimer	6.25	577	289	280
15	Epicatechin	7	289	245; 205	279
16	Kaempferol-3-O-rutinoside	10	593	285	347
17	Isorhamnetin-3-O-rutinoside	10.2	623	315	346
18	Isorhamnetin-3-O-glucoside	10.3	477	315	346
19	Kaempferol-3-O-glucoside	10	447	285	347
20	Rutin	10.9	609	301	350

**Table 3:** Content (mg/kg of almond) of the main phenolic compounds in resin-obtained extracts (ROE) and freeze-dried extracts (FDE).

	ROE	FDE
Chlorogenic acid	0.876 ± 0.021	0.847 ± 0.036
Catechin	30.973 ± 0.677	15.101 ± 0.487
4-p-Coumaroylquinic acid	0.436 ± 0.021	0.428 ± 0.028
Epicatechin	15.224 ± 0.151	6.960 ± 0.241
Epicatechin digallate	2.352 ± 0.138	1.218 ± 0.129
Isorhamnetin-3-O-rutinoside	12.473 ± 0.393	5.575 ± 0.285
Kaempferol-3-O-glucoside	2.704 ± 0.102	1.643 ± 0.020
Kaempferol-3-O-rutinoside	4.852 ± 0.245	2.684 ± 0.050
Vanillic acid dihexose	3.834 ± 0.811	4.787 ± 0.167
Vanillic acid hexose	n.d.	1.955 ± 0.111
Procyanidins	640.398 ± 30.993	347.527 ± 7.044
TOTAL	713.963 ± 32.443	388.724 ± 6.496

n.d. = not detected.

## Data Availability

No data were used to support this study.

## Supplementary Materials

Supplementary Materials Figure S1: graphical illustration of the experiment's design. This figure summarizes all the experiments conducted and detailed in the abstract. After blanching and draining the almonds, the resulting blanching water is centrifuged, filtrated, and adsorbed on the resin by counterflow. Desorption is performed by downflow using acetone. The resulting fractions are evaporated until a dry extract is obtained. The antimicrobial and antioxidant activities of this extract have been tested and examples of application (encapsulation) have been provided. Figure S2: adsorption apparatus. This figure illustrates an XK 50 double-jacket glass column (England) with a length of 30 cm and an internal diameter of 5 cm. The column was filled with 150 g of preprepared resin to a height of 16 cm. The adsorption was performed by upward flow using a peristaltic pump (red box). The system is connected with a nitrogen inlet (needle) to limit the oxidation of polyphenols. Figure S3: closing of the chalcone nucleus. The chalcone nuclei of flavonoids naturally present in almond skin spontaneously equilibrates after 2 hours in the adsorption column and undergo cyclization under neutral conditions, increasing in flavonoid concentration.
