# Supplementary material for: Adsorption of Polyphenols from Almond Blanching Water by Macroporous Resin
Source: Int J Food Sci. 2022 Jun 3;2022:7847276. doi: 10.1155/2022/7847276 (PMC9277471; doi:10.1155/2022/7847276)
Supplement: Supplementary Materials — Figure S1: graphical illustration of the experiment's design. This figure summarizes all the experiments conducted and detailed in the abstract. After blanching and draining the almonds, the resulting blanching water is centrifuged, filtrated, and adsorbed on the resin by counterflow. Desorption is performed by downflow using acetone. The resulting fractions are evaporated until a dry extract is obtained. The antimicrobial and antioxidant activities of this extract have been tested and examples of application (encapsulation) have been provided. Figure S2: adsorption apparatus. This figure illustrates an XK 50 double-jacket glass column (England) with a length of 30 cm and an internal diameter of 5 cm. The column was filled with 150 g of preprepared resin to a height of 16 cm. The adsorption was performed by upward flow using a peristaltic pump (red box). The system is connected with a nitrogen inlet (needle) to limit the oxidation of polyphenols. Figure S3: closing of the chalcone nucleus. The chalcone nuclei of flavonoids naturally present in almond skin spontaneously equilibrates after 2 hours in the adsorption column and undergo cyclization under neutral conditions, increasing in flavonoid concentration. [file 7847276.f1.docx]

**Adsorption of polyphenols from almond blanching water by macroporous resin – Supplementary Materials**

**
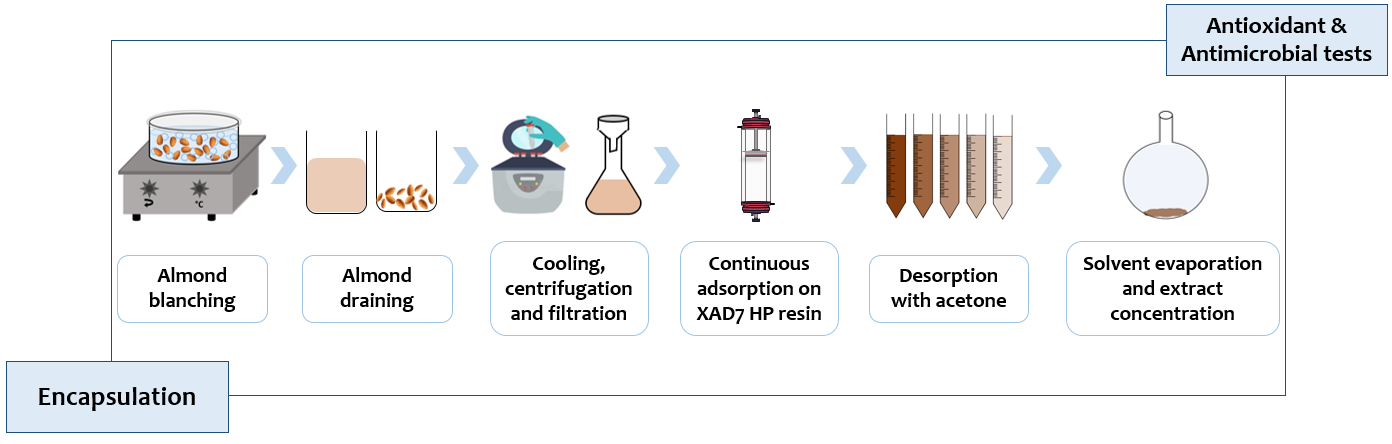
**


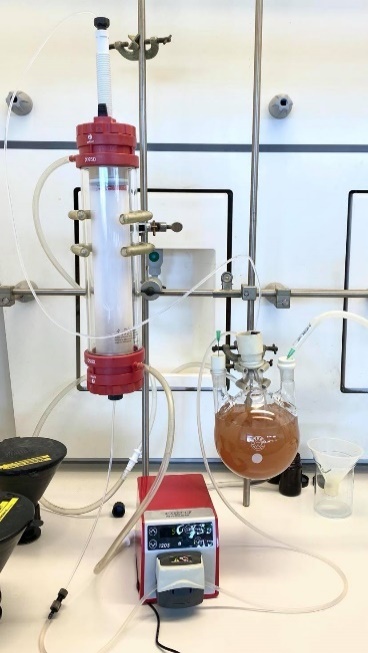
 **Figure S1.** Graphical illustration of the experiment’s design


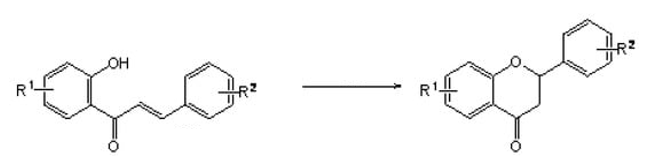
**Figure S2**. Adsorption apparatus

**Figure S3.** Closing of the chalcone nucleus
